# 噬血细胞综合征合并甲胎蛋白升高3例报告及文献复习

**DOI:** 10.3760/cma.j.issn.0253-2727.2023.12.013

**Published:** 2023-12

**Authors:** 玉梅 刘, 薇 张, 秋颖 曹, 忻琰 谢, 丽娟 李, 蓉 付, 宗鸿 邵, 嘉 宋

**Affiliations:** 天津医科大学总医院血液科，天津 300052 Department of Hematology, Tianjin Medical University General Hospital, Tianjin 300052, China

噬血细胞综合征（HPS），又称为噬血细胞性淋巴组织细胞增多症（HLH），是一种免疫调节功能异常引起的严重炎症反应综合征，可致多器官系统损伤，甚至危及生命，预后较差。肝功能异常（主要为转氨酶水平升高）存在于75％的成人和儿童HPS中[1]。甲胎蛋白（AFP）属于蛋白类基因家族，在胎儿肝脏中表达相对较高，特别是在先天性异常的胎儿肝脏中[2]–[3]。血清AFP水平在出生后几周内下降，并保持较低水平。成人AFP升高发生在肝癌和肝脏再生过程中[4]。HPS合并AFP升高的病例未见报道，本研究回顾性分析了HPS合并AFP升高患者的临床特点，并进行文献复习，以提高对本病的认识水平。

## 病例与方法

1. 病例：收集2019年11月至2023年2月天津医科大学总医院收治的54例诊断HPS同时治疗前检测了AFP水平的患者临床资料，并进行回顾性分析。所有患者均符合HLH 2004诊断标准，其中包括3例治疗前AFP升高的患者。临床病例资料包括患者的发病经过、症状体征、辅助检查结果、治疗方案、疗效评估及预后。

2. 疗效评价及随访：疗效评价标准参照《中国噬血细胞综合征诊断与治疗指南（2022版）》[5]，分为完全缓解（CR）、部分缓解（PR）和无效（NR）。电话随访患者的预后，随访时间截至2023年3月31日。

## 结果

1. 3例AFP升高患者的临床资料：3例HPS合并AFP升高的患者均为年轻女性，均经影像学检查排除了肿瘤。其中1例AFP水平显著升高者（例1，AFP>2 000 ng/ml），为原因不明的HPS；2例AFP轻度升高者（例2和3，AFP<50 ng/ml），原发病均为系统性红斑狼疮（SLE）。3例患者均存在显著的肝功能异常，主要表现为转氨酶水平显著升高伴有高胆红素血症及低白蛋白血症（表1）。治疗方案：例1予DEP方案化疗（多柔比星脂质体、依托泊苷、甲泼尼龙）、芦可替尼抗炎及保肝治疗，例2予积极调节免疫治疗原发病、抗炎及保肝治疗，例3予积极调节免疫治疗原发病、抗炎、控制感染及胰腺炎、保肝治疗，依托泊苷治疗HPS。

**表1:** 3例AFP升高的噬血细胞综合征患者的临床及实验室特点

临床特征及检查项目	例 1	例 2	例 3
性别	女	女	女
年龄（岁）	23	11	26
AFP（ng/ml）	>2 000	46.58	26.56
铁蛋白（ng/ml）	10 518	2 764	17 242
Child-Pugh分级	B	B	B
ALB（g/L）	29	26	27
ALT（U/L）	604	580	68
AST（U/L）	744	1 310	1 194
LDH（U/L）	556	859	1 550
TBIL（µmol/L）	210.8	28.7	52.7
DBIL（µmol/L）	165.0	18.3	42.5
WBC（×10^9^/L）	6.59	2.47	6.56
RBC（×10^12^/L）	4.63	5.00	4.35
HGB（g/L）	129	79	105
PLT（×10^9^/L）	72	42	38
尿白蛋白	1+	1+	1+
尿潜血	3+	1+	2+
纤维蛋白原（g/L）	1.35	2.11	1.15
TG（mmol/L）	2.94	3.14	2.03
sCD25（pg/ml）	12 489	NA	7 780
NK细胞活性	正常	NA	正常
吞噬现象	有	有	有
基因异常	未检出	NA	NA
肿瘤	无	无	无
自身免疫疾病	无	SLE	SLE
肺炎	无	无	有
病毒	未检出	EB病毒（+）^a^	未检出
肺炎支原体	未检出	未检出	阳性
结核	未检出	未检出	未检出
其他病原体	未检出	未检出	热带假丝酵母，霉菌
脑脊液	正常	正常	正常
甲状腺功能	正常	正常	FT3：1.99 pmol/L, FT4：13.65 pmol/L, TSH：0.205 mIU/L

注 AFP：甲胎蛋白；ALB：白蛋白；TBIL：总胆红素；DBIL：直接胆红素；TG：甘油三酯；SLE：系统性红斑狼疮；FT3：游离三碘甲状腺原氨酸；FT4：游离甲状腺素；TSH：促甲状腺激素；NA：未检测；病毒：包括EB病毒、巨细胞病毒、人类细小病毒B19、汉坦病毒（流行性出血热）、单纯疱疹病毒1/2、带状疱疹病毒、柯萨奇病毒、风疹病毒、乙型/丙型肝炎病毒以及人类免疫缺陷病毒（HIV）；其他病原体：包括布氏杆菌、伤寒沙门菌、立克次体、弓形虫以及梅毒螺旋体；^a^ EB病毒DNA定量：6.95×10^3^拷贝/ml（正常值：<5×10^3^拷贝/ml）

2. AFP水平与肝功能变化特点：54例HPS患者中肝功能异常患者49例（90.7％），包括肝酶学指标及胆红素水平升高，其余5例患者合并低白蛋白血症。3例AFP升高者在发现AFP升高前均已出现肝功能显著异常，例1在HPS进展期（铁蛋白水平持续升高），随着肝酶学指标升高，AFP水平持续升高，经治疗肝酶学指标有所好转后，AFP仍处于峰值（>2 000 ng/ml），例2和3治疗初期未连续监测AFP水平。3例患者均在原发病及HPS治疗好转后肝功能及AFP水平逐渐恢复正常。

3. 临床转归及预后：54例患者中无法评价疗效者3例，余51例患者中达CR/ PR 者28例（54.9％）。3例AFP升高患者经积极治疗后均存活，例1获得 CR，例2和3原发病病情稳定［SLE疾病活动度评分（SLEDAI）≤4分］且HPS达PR。死亡患者21例，其中6例死于原发病伴HPS，9例死于原发病进展，3例死于HPS，另3例死于其他原因（严重感染2例，脑出血1例）。HPS治疗NR者2例。死亡及NR患者中16例原发病未控制或未治疗原发病（59.4％）。

## 讨论及文献复习

肝生化检查异常在HPS患者中很常见，是HPS诊断的支持证据[6]。HPS肝损伤主要包括肝细胞损伤、胆汁淤积性损伤和混合损伤，可表现为肝肿大、胆汁淤积甚至暴发性肝衰竭。患者肝功能异常的特点主要表现为相关酶学改变、低白蛋白血症、凝血功能异常和高胆红素血症。Kerguenec等[7]的一项研究分析了30例HPS伴肝损伤患者的临床及病理资料，患者中位血清ALT值为正常上限的5倍，中位血清ALP值为正常上限的2.7倍，中位总胆红素值为136（4～681）mmol/L，而血清胆红素高、血清ALP活性升高、基础疾病缺乏治疗与预后不良相关。患者肝脏病理改变为非特异性改变，所有患者的肝活检病理均可见肝窦扩张及吞噬性组织细胞增多。唐伟萍等[8]分析了92例HPS肝损伤患者的临床特点，患者的肝功能异常以轻、中度为主，主要表现为血清LDH（89.13％）和AST（64.13％）升高，白蛋白（90.22％）下降，胆红素升高以直接胆红素（59.78％）为主，APTT（76.09％）以及PT（67.39％）延长，其中LDH≥2 000 U/L、白蛋白<30 g/L和PT≥15.1 s的患者预后较差。本研究HPS患者中合并肝酶学指标和（或）胆红素水平升高患者的比例为90.7％，其余患者存在低白蛋白血症。HPS肝损伤考虑为炎症细胞的浸润以及细胞因子过度产生共同作用的结果。本研究中，AFP升高3例患者表现为更严重的肝损伤，尤其是转氨酶水平显著升高，提示AFP水平可能与肝损伤程度相关。

血清AFP水平是肝细胞癌的生物标志物，AFP≥100 ng/ml对诊断早期肝细胞癌特异性可达99％，但敏感性仅31％[9]。血清AFP水平升高也可发生在某些非恶性疾病（如慢性乙型或丙型病毒性肝炎）患者中，并在急性肝衰竭（ALF）中较常见[3],[10]。因此专家小组认为，AFP水平升高需联合影像学阳性发现诊断肝细胞癌[11]。而血清AFP水平升高在HPS中较罕见。本研究中的3例HPS合并AFP水平升高的患者均经影像学检查排除了肝癌的诊断。

AFP升高被认为是肝去分化再生的标志。研究认为AFP是肝祖细胞分泌的，在成熟肝细胞中不表达，而肝祖细胞是参与肝再生的主要细胞[12]。因此，急性肝损伤（ALI）伴广泛坏死后，肝细胞再生时AFP可升高[3],[13]。Kakisaka等[14]的研究也证明血清AFP水平可能是肝祖细胞诱导和增殖的标志物。ALI/ALF患者的血清AFP水平显著升高，且与肝脏中细胞角蛋白CK-7阳性肝祖细胞数量显著相关（严重受损肝脏除外）。因此，血清AFP水平可反映ALI/ALF患者肝祖细胞的诱导活化水平。而ALF死亡患者的肝祖细胞增殖受损，AFP峰值提前，成熟肝细胞产生不足。研究发现AFP可与肝细胞生长因子受体等多种增殖因子结合，增强其表达，促进肝细胞增殖、分化和再生。AFP还可减轻肿瘤坏死因子-α介导的肝损伤，促进肝细胞增殖[15]。此外，AFP可作为维持血浆胶体渗透压的载体，运输胆红素、脂肪酸和类固醇等，调节稳态[16]。因此，AFP在促进肝再生过程中起重要作用。

Yang等[4]的研究显示，ALF患者治疗3 d后AFP水平较治疗开始时升高与患者的预后良好相关，AFP水平的下降是此类患者预后不良的标志。Schiødt等[3]的一项前瞻性研究也表明，入院时AFP的绝对值较高并不能预测ALF患者的良好预后，而在入院第1天和第3天之间AFP值的升高表明预后更好，非肝移植自发幸存者的AFP比值（入院第3天AFP值与第1天之比）为2.20（0.11～22.10），非幸存者的AFP比值为0.87（0.11～16.40）（*P*<0.001）。在98例幸存者中观察到70例（71％）AFP比值>1，而在64例非幸存者中观察到51例（80％）AFP比值<1。此外，Schmidt等[13]的一项关于对乙酰氨基酚诱导的肝损伤患者的前瞻性研究表明，AFP水平高于20 ng/mL与良好预后相关。在儿童患者中的研究也得到与成人相似的结论[17]。本研究中3例HPS合并AFP水平升高患者，1例在治疗初期AFP水平持续升高，2例因未连续监测AFP水平，未发现此趋势。3例患者经积极治疗原发病及抗炎、化疗治疗HPS后均获得良好预后。

综上所述，HPS合并AFP升高病例罕见，临床表现无特异性，多合并肝损伤，肝酶学水平明显升高伴有高胆红素血症及低白蛋白血症，治疗以治疗原发病和HPS为主，辅助保肝、控制并发症、支持治疗。本研究HPS伴AFP升高病例数少，尚无法得出AFP升高对HPS患者预后的影响，今后尚需累积病例数分析预后趋势，并从分子水平进一步研究HPS中AFP升高的机制。
